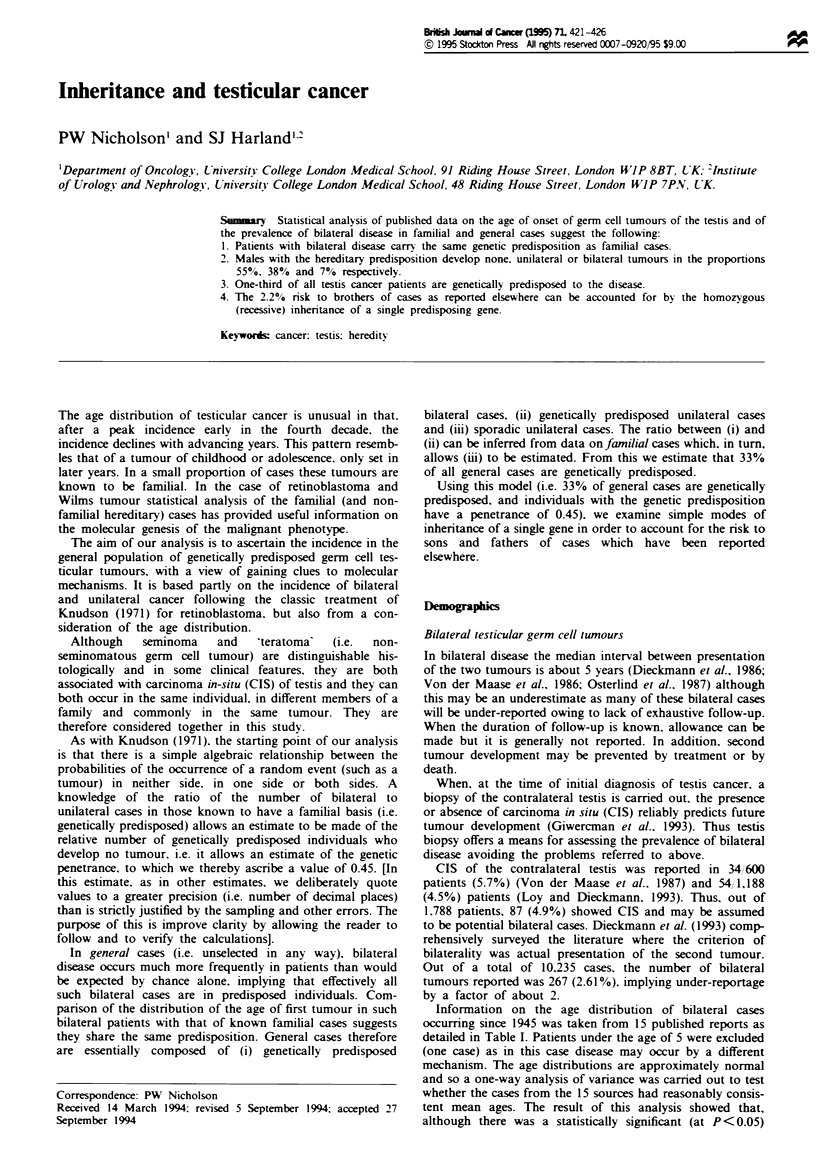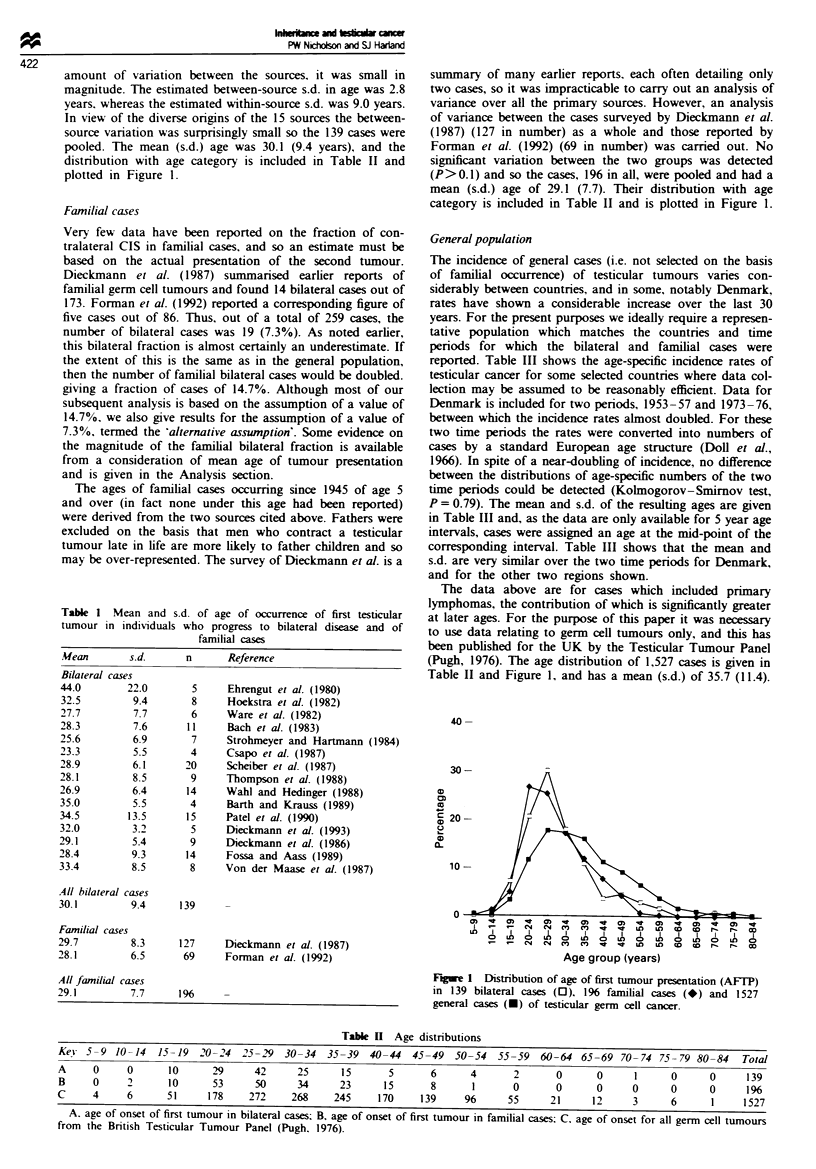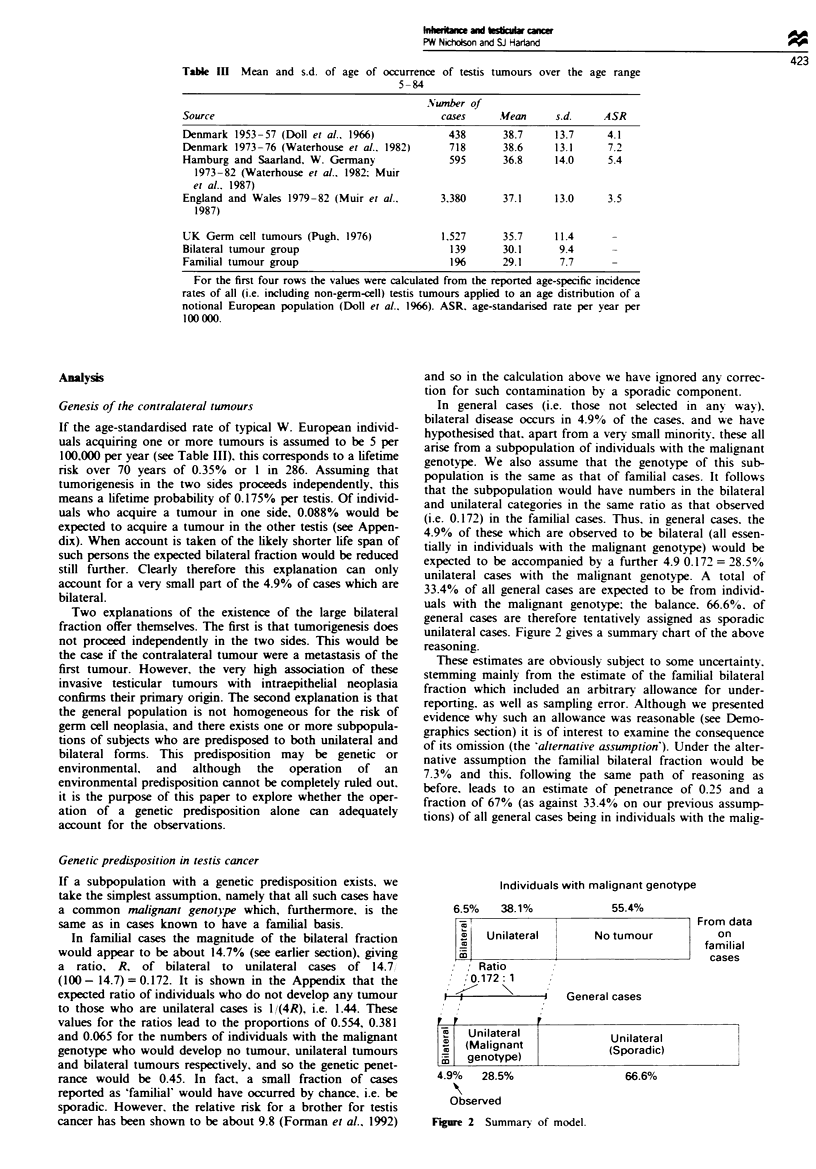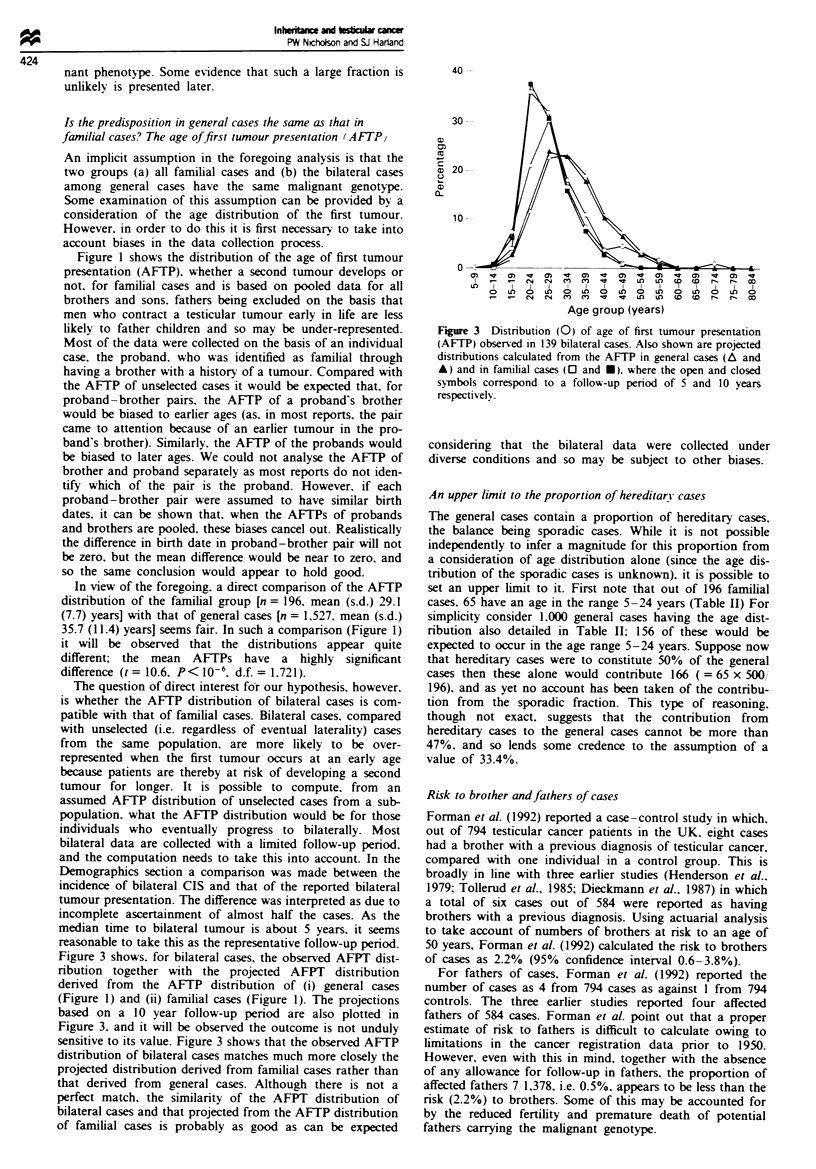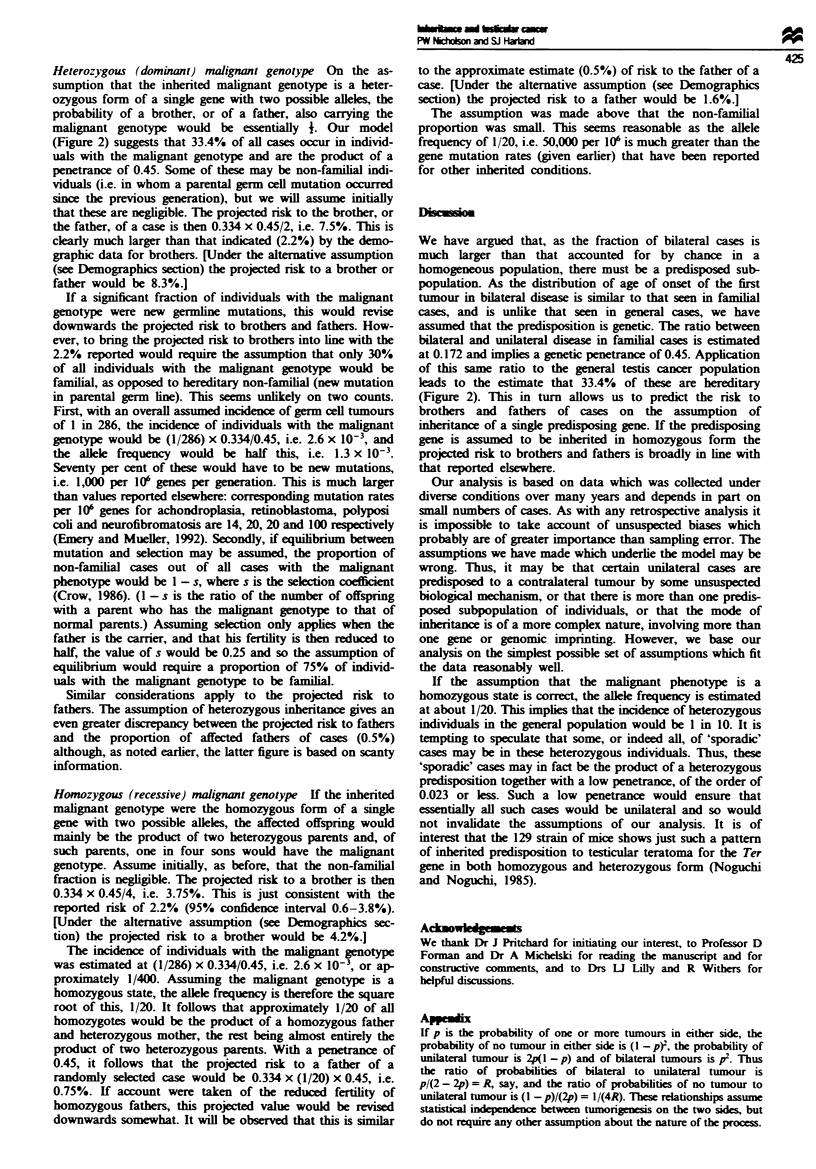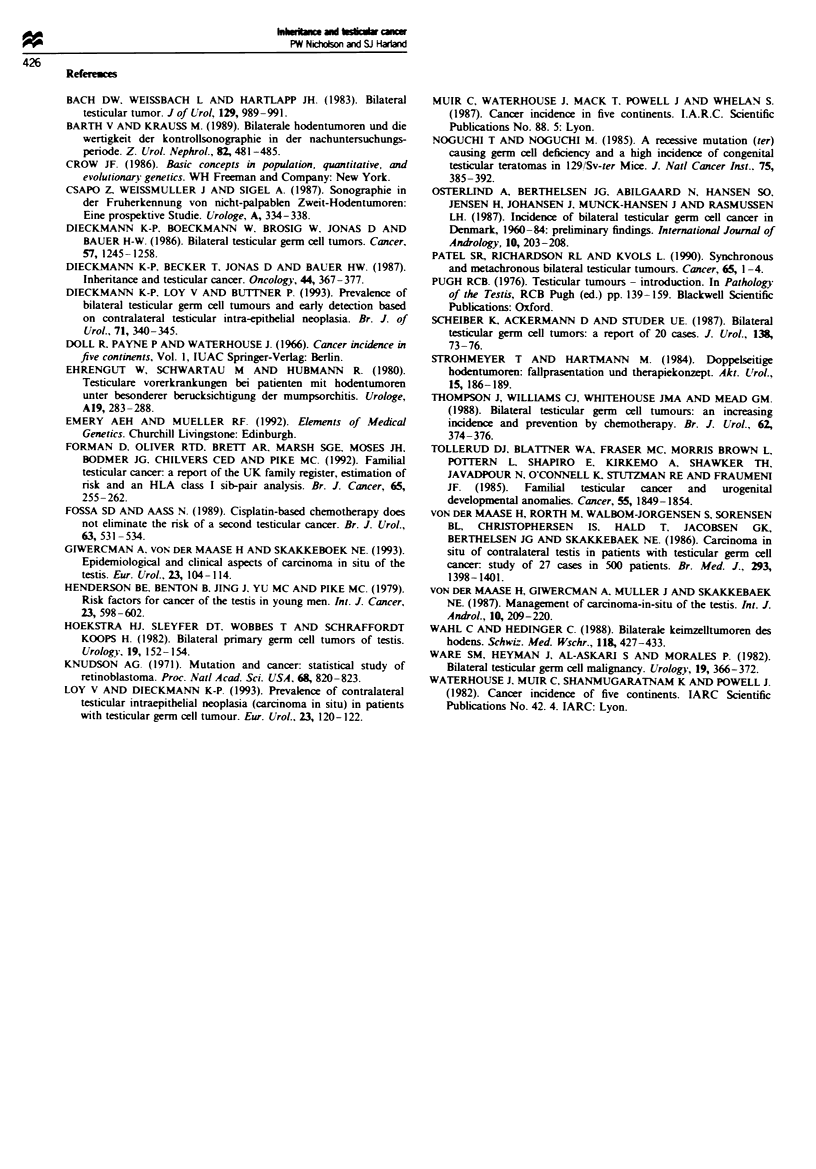# Inheritance and testicular cancer.

**DOI:** 10.1038/bjc.1995.86

**Published:** 1995-02

**Authors:** P. W. Nicholson, S. J. Harland

**Affiliations:** Department of Oncology, University College London Medical School, UK.

## Abstract

Statistical analysis of published data on the age of onset of germ cell tumours of the testis and of the prevalence of bilateral disease in familial and general cases suggest the following: 1. Patients with bilateral disease carry the same genetic predisposition as familial cases. 2. Males with the hereditary predisposition develop none, unilateral or bilateral tumours in the proportions 55%, 38% and 7% respectively. 3. One-third of all testis cancer patients are genetically predisposed to the disease. 4. The 2.2% risk to brothers of cases as reported elsewhere can be accounted for by the homozygous (recessive) inheritance of a single predisposing gene.


British Journal of Cancer (1995) 71, 421-426

© 1995 Stockton Press All rights reserved 0007-0920/95 $9.00

### Inheritance and testicular cancer

PW Nicholson[1] and SJ Harland[1,2]

[1]*Department of Oncology, University College London Medical School, 91 Riding House Street, London W1P 8BT, UK; [2]Institute of Urology and Nephrology, University College London Medical School, 48 Riding House Street, London W1P 7PN, UK.*

**Summary** Statistical analysis of published data on the age of onset of germ cell tumours of the testis and of the prevalence of bilateral disease in familial and general cases suggest the following:
1. Patients with bilateral disease carry the same genetic predisposition as familial cases.
2. Males with the hereditary predisposition develop none, unilateral or bilateral tumours in the proportions 55%, 38% and 7% respectively.
3. One-third of all testis cancer patients are genetically predisposed to the disease.
4. The 2.2% risk to brothers of cases as reported elsewhere can be accounted for by the homozygous (recessive) inheritance of a single predisposing gene.

**Keywords:** cancer; testis; heredity

The age distribution of testicular cancer is unusual in that, after a peak incidence early in the fourth decade, the incidence declines with advancing years. This pattern resembles that of a tumour of childhood or adolescence, only set in later years. In a small proportion of cases these tumours are known to be familial. In the case of retinoblastoma and Wilms tumour statistical analysis of the familial (and non-familial hereditary) cases has provided useful information on the molecular genesis of the malignant phenotype.

The aim of our analysis is to ascertain the incidence in the general population of genetically predisposed germ cell testicular tumours, with a view of gaining clues to molecular mechanisms. It is based partly on the incidence of bilateral and unilateral cancer following the classic treatment of Knudson (1971) for retinoblastoma, but also from a consideration of the age distribution.

Although seminoma and 'teratoma' (i.e. non-seminomatous germ cell tumour) are distinguishable histologically and in some clinical features, they are both associated with carcinoma *in-situ* (CIS) of testis and they can both occur in the same individual, in different members of a family and commonly in the same tumour. They are therefore considered together in this study.

As with Knudson (1971), the starting point of our analysis is that there is a simple algebraic relationship between the probabilities of the occurrence of a random event (such as a tumour) in neither side, in one side or both sides. A knowledge of the ratio of the number of bilateral to unilateral cases in those known to have a familial basis (i.e. genetically predisposed) allows an estimate to be made of the relative number of genetically predisposed individuals who develop no tumour, i.e. it allows an estimate of the genetic penetrance, to which we thereby ascribe a value of 0.45. [In this estimate, as in other estimates, we deliberately quote values to a greater precision (i.e. number of decimal places) than is strictly justified by the sampling and other errors. The purpose of this is improve clarity by allowing the reader to follow and to verify the calculations].

In *general* cases (i.e. unselected in any way), bilateral disease occurs much more frequently in patients than would be expected by chance alone, implying that effectively all such bilateral cases are in predisposed individuals. Comparison of the distribution of the age of first tumour in such bilateral patients with that of known familial cases suggests they share the same predisposition. General cases therefore are essentially composed of (i) genetically predisposed bilateral cases, (ii) genetically predisposed unilateral cases and (iii) sporadic unilateral cases. The ratio between (i) and (ii) can be inferred from data on *familial* cases which, in turn, allows (iii) to be estimated. From this we estimate that 33% of all general cases are genetically predisposed.

Using this model (i.e. 33% of general cases are genetically predisposed, and individuals with the genetic predisposition have a penetrance of 0.45), we examine simple modes of inheritance of a single gene in order to account for the risk to sons and fathers of cases which have been reported elsewhere.

### Demographics

### *Bilateral testicular germ cell tumours*

In bilateral disease the median interval between presentation of the two tumours is about 5 years (Dieckmann *et al.*, 1986; Von der Maase *et al.*, 1986; Osterlind *et al.*, 1987) although this may be an underestimate as many of these bilateral cases will be under-reported owing to lack of exhaustive follow-up. When the duration of follow-up is known, allowance can be made but it is generally not reported. In addition, second tumour development may be prevented by treatment or by death.

When, at the time of initial diagnosis of testis cancer, a biopsy of the contralateral testis is carried out, the presence or absence of carcinoma *in situ* (CIS) reliably predicts future tumour development (Giwercman *et al.*, 1993). Thus testis biopsy offers a means for assessing the prevalence of bilateral disease avoiding the problems referred to above.

CIS of the contralateral testis was reported in 34/600 patients (5.7%) (Von der Maase *et al.*, 1987) and 54/1,188 (4.5%) patients (Loy and Dieckmann, 1993). Thus, out of 1,788 patients, 87 (4.9%) showed CIS and may be assumed to be potential bilateral cases. Dieckmann *et al.* (1993) comprehensively surveyed the literature where the criterion of bilaterality was actual presentation of the second tumour. Out of a total of 10,235 cases, the number of bilateral tumours reported was 267 (2.61%), implying under-reportage by a factor of about 2.

Information on the age distribution of bilateral cases occurring since 1945 was taken from 15 published reports as detailed in Table I. Patients under the age of 5 were excluded (one case) as in this case disease may occur by a different mechanism. The age distributions are approximately normal and so a one-way analysis of variance was carried out to test whether the cases from the 15 sources had reasonably consistent mean ages. The result of this analysis showed that, although there was a statistically significant (at $P<0.05$)

Correspondence: PW Nicholson
Received 14 March 1994; revised 5 September 1994; accepted 27 September 1994

amount of variation between the sources, it was small in magnitude. The estimated between-source s.d. in age was 2.8 years, whereas the estimated within-source s.d. was 9.0 years. In view of the diverse origins of the 15 sources the between-source variation was surprisingly small so the 139 cases were pooled. The mean (s.d.) age was 30.1 (9.4 years), and the distribution with age category is included in Table II and plotted in Figure 1.

### *Familial cases*

Very few data have been reported on the fraction of contralateral CIS in familial cases, and so an estimate must be based on the actual presentation of the second tumour. Dieckmann *et al.* (1987) summarised earlier reports of familial germ cell tumours and found 14 bilateral cases out of 173. Forman *et al.* (1992) reported a corresponding figure of five cases out of 86. Thus, out of a total of 259 cases, the number of bilateral cases was 19 (7.3%). As noted earlier, this bilateral fraction is almost certainly an underestimate. If the extent of this is the same as in the general population, then the number of familial bilateral cases would be doubled, giving a fraction of cases of 14.7%. Although most of our subsequent analysis is based on the assumption of a value of 14.7%, we also give results for the assumption of a value of 7.3%, termed the '*alternative assumption*'. Some evidence on the magnitude of the familial bilateral fraction is available from a consideration of mean age of tumour presentation and is given in the Analysis section.

The ages of familial cases occurring since 1945 of age 5 and over (in fact none under this age had been reported) were derived from the two sources cited above. Fathers were excluded on the basis that men who contract a testicular tumour late in life are more likely to father children and so may be over-represented. The survey of Dieckmann *et al.* is a summary of many earlier reports, each often detailing only two cases, so it was impracticable to carry out an analysis of variance over all the primary sources. However, an analysis of variance between the cases surveyed by Dieckmann *et al.* (1987) (127 in number) as a whole and those reported by Forman *et al.* (1992) (69 in number) was carried out. No significant variation between the two groups was detected ($P>0.1$) and so the cases, 196 in all, were pooled and had a mean (s.d.) age of 29.1 (7.7). Their distribution with age category is included in Table II and is plotted in Figure 1.

**Table I** Mean and s.d. of age of occurrence of first testicular tumour in individuals who progress to bilateral disease and of familial cases

| *Mean* | *s.d.* | n | *Reference* |
|---|---|---|---|
| *Bilateral cases* | | | |
| 44.0 | 22.0 | 5 | Ehrengut *et al.* (1980) |
| 32.5 | 9.4 | 8 | Hoekstra *et al.* (1982) |
| 27.7 | 7.7 | 6 | Ware *et al.* (1982) |
| 28.3 | 7.6 | 11 | Bach *et al.* (1983) |
| 25.6 | 6.9 | 7 | Strohmeyer and Hartmann (1984) |
| 23.3 | 5.5 | 4 | Csapo *et al.* (1987) |
| 28.9 | 6.1 | 20 | Scheiber *et al.* (1987) |
| 28.1 | 8.5 | 9 | Thompson *et al.* (1988) |
| 26.9 | 6.4 | 14 | Wahl and Hedinger (1988) |
| 35.0 | 5.5 | 4 | Barth and Krauss (1989) |
| 34.5 | 13.5 | 15 | Patel *et al.* (1990) |
| 32.0 | 3.2 | 5 | Dieckmann *et al.* (1993) |
| 29.1 | 5.4 | 9 | Dieckmann *et al.* (1986) |
| 28.4 | 9.3 | 14 | Fossa and Aass (1989) |
| 33.4 | 8.5 | 8 | Von der Maase *et al.* (1987) |
| *All bilateral cases* | | | |
| 30.1 | 9.4 | 139 | – |
| *Familial cases* | | | |
| 29.7 | 8.3 | 127 | Dieckmann *et al.* (1987) |
| 28.1 | 6.5 | 69 | Forman *et al.* (1992) |
| *All familial cases* | | | |
| 29.1 | 7.7 | 196 | – |

### *General population*

The incidence of general cases (i.e. not selected on the basis of familial occurrence) of testicular tumours varies considerably between countries, and in some, notably Denmark, rates have shown a considerable increase over the last 30 years. For the present purposes we ideally require a representative population which matches the countries and time periods for which the bilateral and familial cases were reported. Table III shows the age-specific incidence rates of testicular cancer for some selected countries where data collection may be assumed to be reasonably efficient. Data for Denmark is included for two periods, 1953–57 and 1973–76, between which the incidence rates almost doubled. For these two time periods the rates were converted into numbers of cases by a standard European age structure (Doll *et al.*, 1966). In spite of a near-doubling of incidence, no difference between the distributions of age-specific numbers of the two time periods could be detected (Kolmogorov–Smirnov test, $P = 0.79$). The mean and s.d. of the resulting ages are given in Table III and, as the data are only available for 5 year age intervals, cases were assigned an age at the mid-point of the corresponding interval. Table III shows that the mean and s.d. are very similar over the two time periods for Denmark, and for the other two regions shown.

The data above are for cases which included primary lymphomas, the contribution of which is significantly greater at later ages. For the purpose of this paper it was necessary to use data relating to germ cell tumours only, and this has been published for the UK by the Testicular Tumour Panel (Pugh, 1976). The age distribution of 1,527 cases is given in Table II and Figure 1, and has a mean (s.d.) of 35.7 (11.4).

**Figure 1** Distribution of age of first tumour presentation (AFTP) in 139 bilateral cases (□), 196 familial cases (◆) and 1527 general cases (■) of testicular germ cell cancer.

**Table II** Age distributions

| *Key* | *5–9* | *10–14* | *15–19* | *20–24* | *25–29* | *30–34* | *35–39* | *40–44* | *45–49* | *50–54* | *55–59* | *60–64* | *65–69* | *70–74* | *75–79* | *80–84* | *Total* |
|---|---|---|---|---|---|---|---|---|---|---|---|---|---|---|---|---|---|
| A | 0 | 0 | 10 | 29 | 42 | 25 | 15 | 5 | 6 | 4 | 2 | 0 | 0 | 1 | 0 | 0 | 139 |
| B | 0 | 2 | 10 | 53 | 50 | 34 | 23 | 15 | 8 | 1 | 0 | 0 | 0 | 0 | 0 | 0 | 196 |
| C | 4 | 6 | 51 | 178 | 272 | 268 | 245 | 170 | 139 | 96 | 55 | 21 | 12 | 3 | 6 | 1 | 1527 |

A, age of onset of first tumour in bilateral cases; B, age of onset of first tumour in familial cases; C, age of onset for all germ cell tumours from the British Testicular Tumour Panel (Pugh, 1976).

**Table III** Mean and s.d. of age of occurrence of testis tumours over the age range 5–84

| Source | Number of cases | Mean | s.d. | ASR |
|---|---|---|---|---|
| Denmark 1953–57 (Doll *et al*., 1966) | 438 | 38.7 | 13.7 | 4.1 |
| Denmark 1973–76 (Waterhouse *et al*., 1982) | 718 | 38.6 | 13.1 | 7.2 |
| Hamburg and Saarland, W. Germany 1973–82 (Waterhouse *et al*., 1982; Muir *et al*., 1987) | 595 | 36.8 | 14.0 | 5.4 |
| England and Wales 1979–82 (Muir *et al*., 1987) | 3,380 | 37.1 | 13.0 | 3.5 |
| UK Germ cell tumours (Pugh, 1976) | 1,527 | 35.7 | 11.4 | – |
| Bilateral tumour group | 139 | 30.1 | 9.4 | – |
| Familial tumour group | 196 | 29.1 | 7.7 | – |

For the first four rows the values were calculated from the reported age-specific incidence rates of all (i.e. including non-germ-cell) testis tumours applied to an age distribution of a notional European population (Doll *et al*., 1966). ASR, age-standarised rate per year per 100 000.

### Analysis

### *Genesis of the contralateral tumours*

If the age-standardised rate of typical W. European individuals acquiring one or more tumours is assumed to be 5 per 100,000 per year (see Table III), this corresponds to a lifetime risk over 70 years of 0.35% or 1 in 286. Assuming that tumorigenesis in the two sides proceeds independently, this means a lifetime probability of 0.175% per testis. Of individuals who acquire a tumour in one side, 0.088% would be expected to acquire a tumour in the other testis (see Appendix). When account is taken of the likely shorter life span of such persons the expected bilateral fraction would be reduced still further. Clearly therefore this explanation can only account for a very small part of the 4.9% of cases which are bilateral.

Two explanations of the existence of the large bilateral fraction offer themselves. The first is that tumorigenesis does not proceed independently in the two sides. This would be the case if the contralateral tumour were a metastasis of the first tumour. However, the very high association of these invasive testicular tumours with intraepithelial neoplasia confirms their primary origin. The second explanation is that the general population is not homogeneous for the risk of germ cell neoplasia, and there exists one or more subpopulations of subjects who are predisposed to both unilateral and bilateral forms. This predisposition may be genetic or environmental, and although the operation of an environmental predisposition cannot be completely ruled out, it is the purpose of this paper to explore whether the operation of a genetic predisposition alone can adequately account for the observations.

### *Genetic predisposition in testis cancer*

If a subpopulation with a genetic predisposition exists, we take the simplest assumption, namely that all such cases have a common *malignant genotype* which, furthermore, is the same as in cases known to have a familial basis.

In familial cases the magnitude of the bilateral fraction would appear to be about 14.7% (see earlier section), giving a ratio, $R$, of bilateral to unilateral cases of 14.7/(100 – 14.7) = 0.172. It is shown in the Appendix that the expected ratio of individuals who do not develop any tumour to those who are unilateral cases is $1/(4R)$, i.e. 1.44. These values for the ratios lead to the proportions of 0.554, 0.381 and 0.065 for the numbers of individuals with the malignant genotype who would develop no tumour, unilateral tumours and bilateral tumours respectively, and so the genetic penetrance would be 0.45. In fact, a small fraction of cases reported as 'familial' would have occurred by chance, i.e. be sporadic. However, the relative risk for a brother for testis cancer has been shown to be about 9.8 (Forman *et al*., 1992) and so in the calculation above we have ignored any correction for such contamination by a sporadic component.

In general cases (i.e. those not selected in any way), bilateral disease occurs in 4.9% of the cases, and we have hypothesised that, apart from a very small minority, these all arise from a subpopulation of individuals with the malignant genotype. We also assume that the genotype of this subpopulation is the same as that of familial cases. It follows that the subpopulation would have numbers in the bilateral and unilateral categories in the same ratio as that observed (i.e. 0.172) in the familial cases. Thus, in general cases, the 4.9% of these which are observed to be bilateral (all essentially in individuals with the malignant genotype) would be expected to be accompanied by a further 4.9 0.172 = 28.5% unilateral cases with the malignant genotype. A total of 33.4% of all general cases are expected to be from individuals with the malignant genotype; the balance, 66.6%, of general cases are therefore tentatively assigned as sporadic unilateral cases. Figure 2 gives a summary chart of the above reasoning.

These estimates are obviously subject to some uncertainty, stemming mainly from the estimate of the familial bilateral fraction which included an arbitrary allowance for under-reporting, as well as sampling error. Although we presented evidence why such an allowance was reasonable (see Demographics section) it is of interest to examine the consequence of its omission (the '*alternative assumption*'). Under the alternative assumption the familial bilateral fraction would be 7.3% and this, following the same path of reasoning as before, leads to an estimate of penetrance of 0.25 and a fraction of 67% (as against 33.4% on our previous assumptions) of all general cases being in individuals with the malig-

**Figure 2** Summary of model.

nant phenotype. Some evidence that such a large fraction is unlikely is presented later.

*Is the predisposition in general cases the same as that in familial cases? The age of first tumour presentation (AFTP)*

An implicit assumption in the foregoing analysis is that the two groups (a) all familial cases and (b) the bilateral cases among general cases have the same malignant genotype. Some examination of this assumption can be provided by a consideration of the age distribution of the first tumour. However, in order to do this it is first necessary to take into account biases in the data collection process.

Figure 1 shows the distribution of the age of first tumour presentation (AFTP), whether a second tumour develops or not, for familial cases and is based on pooled data for all brothers and sons, fathers being excluded on the basis that men who contract a testicular tumour early in life are less likely to father children and so may be under-represented. Most of the data were collected on the basis of an individual case, the proband, who was identified as familial through having a brother with a history of a tumour. Compared with the AFTP of unselected cases it would be expected that, for proband–brother pairs, the AFTP of a proband's brother would be biased to earlier ages (as, in most reports, the pair came to attention because of an earlier tumour in the proband's brother). Similarly, the AFTP of the probands would be biased to later ages. We could not analyse the AFTP of brother and proband separately as most reports do not identify which of the pair is the proband. However, if each proband–brother pair were assumed to have similar birth dates, it can be shown that, when the AFTPs of probands and brothers are pooled, these biases cancel out. Realistically the difference in birth date in proband–brother pair will not be zero, but the mean difference would be near to zero, and so the same conclusion would appear to hold good.

In view of the foregoing, a direct comparison of the AFTP distribution of the familial group [$n = 196$, mean (s.d.) 29.1 (7.7) years] with that of general cases [$n = 1,527$, mean (s.d.) 35.7 (11.4) years] seems fair. In such a comparison (Figure 1) it will be observed that the distributions appear quite different; the mean AFTPs have a highly significant difference ($t = 10.6$, $P < 10^{-6}$, d.f. = 1,721).

The question of direct interest for our hypothesis, however, is whether the AFTP distribution of bilateral cases is compatible with that of familial cases. Bilateral cases, compared with unselected (i.e. regardless of eventual laterality) cases from the same population, are more likely to be over-represented when the first tumour occurs at an early age because patients are thereby at risk of developing a second tumour for longer. It is possible to compute, from an assumed AFTP distribution of unselected cases from a subpopulation, what the AFTP distribution would be for those individuals who eventually progress to bilaterally. Most bilateral data are collected with a limited follow-up period, and the computation needs to take this into account. In the Demographics section a comparison was made between the incidence of bilateral CIS and that of the reported bilateral tumour presentation. The difference was interpreted as due to incomplete ascertainment of almost half the cases. As the median time to bilateral tumour is about 5 years, it seems reasonable to take this as the representative follow-up period. Figure 3 shows, for bilateral cases, the observed AFPT distribution together with the projected AFPT distribution derived from the AFTP distribution of (i) general cases (Figure 1) and (ii) familial cases (Figure 1). The projections based on a 10 year follow-up period are also plotted in Figure 3, and it will be observed the outcome is not unduly sensitive to its value. Figure 3 shows that the observed AFTP distribution of bilateral cases matches much more closely the projected distribution derived from familial cases rather than that derived from general cases. Although there is not a perfect match, the similarity of the AFPT distribution of bilateral cases and that projected from the AFTP distribution of familial cases is probably as good as can be expected

**Figure 3** Distribution (○) of age of first tumour presentation (AFTP) observed in 139 bilateral cases. Also shown are projected distributions calculated from the AFTP in general cases (△ and ▲) and in familial cases (□ and ■), where the open and closed symbols correspond to a follow-up period of 5 and 10 years respectively.

considering that the bilateral data were collected under diverse conditions and so may be subject to other biases.

*An upper limit to the proportion of hereditary cases*

The general cases contain a proportion of hereditary cases, the balance being sporadic cases. While it is not possible independently to infer a magnitude for this proportion from a consideration of age distribution alone (since the age distribution of the sporadic cases is unknown), it is possible to set an upper limit to it. First note that out of 196 familial cases, 65 have an age in the range 5–24 years (Table II) For simplicity consider 1,000 general cases having the age distribution also detailed in Table II; 156 of these would be expected to occur in the age range 5–24 years. Suppose now that hereditary cases were to constitute 50% of the general cases then these alone would contribute 166 ( = 65 × 500/196), and as yet no account has been taken of the contribution from the sporadic fraction. This type of reasoning, though not exact, suggests that the contribution from hereditary cases to the general cases cannot be more than 47%, and so lends some credence to the assumption of a value of 33.4%.

*Risk to brother and fathers of cases*

Forman *et al.* (1992) reported a case–control study in which, out of 794 testicular cancer patients in the UK, eight cases had a brother with a previous diagnosis of testicular cancer, compared with one individual in a control group. This is broadly in line with three earlier studies (Henderson *et al.*, 1979; Tollerud *et al.*, 1985; Dieckmann *et al.*, 1987) in which a total of six cases out of 584 were reported as having brothers with a previous diagnosis. Using actuarial analysis to take account of numbers of brothers at risk to an age of 50 years, Forman *et al.* (1992) calculated the risk to brothers of cases as 2.2% (95% confidence interval 0.6–3.8%).

For fathers of cases, Forman *et al.* (1992) reported the number of cases as 4 from 794 cases as against 1 from 794 controls. The three earlier studies reported four affected fathers of 584 cases. Forman *et al.* point out that a proper estimate of risk to fathers is difficult to calculate owing to limitations in the cancer registration data prior to 1950. However, even with this in mind, together with the absence of any allowance for follow-up in fathers, the proportion of affected fathers 7/1,378, i.e. 0.5%, appears to be less than the risk (2.2%) to brothers. Some of this may be accounted for by the reduced fertility and premature death of potential fathers carrying the malignant genotype.

*Heterozygous (dominant) malignant genotype* On the assumption that the inherited malignant genotype is a heterozygous form of a single gene with two possible alleles, the probability of a brother, or of a father, also carrying the malignant genotype would be essentially $\frac{1}{2}$. Our model (Figure 2) suggests that 33.4% of all cases occur in individuals with the malignant genotype and are the product of a penetrance of 0.45. Some of these may be non-familial individuals (i.e. in whom a parental germ cell mutation occurred since the previous generation), but we will assume initially that these are negligible. The projected risk to the brother, or the father, of a case is then $0.334 \times 0.45$ 2, i.e. 7.5%. This is clearly much larger than that indicated (2.2%) by the demographic data for brothers. [Under the alternative assumption (see Demographics section) the projected risk to a brother or father would be 8.3%.]

If a significant fraction of individuals with the malignant genotype were new germline mutations, this would revise downwards the projected risk to brothers and fathers. However, to bring the projected risk to brothers into line with the 2.2% reported would require the assumption that only 30% of all individuals with the malignant genotype would be familial, as opposed to hereditary non-familial (new mutation in parental germ line). This seems unlikely on two counts. First, with an overall assumed incidence of germ cell tumours of 1 in 286, the incidence of individuals with the malignant genotype would be (1 286) $\times$ 0.334 0.45, i.e. $2.6 \times 10^{-3}$, and the allele frequency would be half this, i.e. $1.3 \times 10^{-3}$. Seventy per cent of these would have to be new mutations, i.e. 1,000 per $10^6$ genes per generation. This is much larger than values reported elsewhere: corresponding mutation rates per $10^6$ genes for achondroplasia, retinoblastoma, polyposi coli and neurofibromatosis are 14, 20, 20 and 100 respectively (Emery and Mueller, 1992). Secondly, if equilibrium between mutation and selection may be assumed, the proportion of non-familial cases out of all cases with the malignant phenotype would be $1 - s$, where $s$ is the selection coefficient (Crow, 1986). ($1 - s$ is the ratio of the number of offspring with a parent who has the malignant genotype to that of normal parents.) Assuming selection only applies when the father is the carrier, and that his fertility is then reduced to half, the value of $s$ would be 0.25 and so the assumption of equilibrium would require a proportion of 75% of individuals with the malignant genotype to be familial.

Similar considerations apply to the projected risk to fathers. The assumption of heterozygous inheritance gives an even greater discrepancy between the projected risk to fathers and the proportion of affected fathers of cases (0.5%) although, as noted earlier, the latter figure is based on scanty information.

*Homozygous (recessive) malignant genotype* If the inherited malignant genotype were the homozygous form of a single gene with two possible alleles, the affected offspring would mainly be the product of two heterozygous parents and, of such parents, one in four sons would have the malignant genotype. Assume initially, as before, that the non-familial fraction is negligible. The projected risk to a brother is then $0.334 \times 0.45$ 4, i.e. 3.75%. This is just consistent with the reported risk of 2.2% (95% confidence interval 0.6–3.8%). [Under the alternative assumption (see Demographics section) the projected risk to a brother would be 4.2%.]

The incidence of individuals with the malignant genotype was estimated at (1 286) $\times$ 0.334 0.45, i.e. $2.6 \times 10^{-3}$, or approximately 1 400. Assuming the malignant genotype is a homozygous state, the allele frequency is therefore the square root of this, 1 20. It follows that approximately 1 20 of all homozygotes would be the product of a homozygous father and heterozygous mother, the rest being almost entirely the product of two heterozygous parents. With a penetrance of 0.45, it follows that the projected risk to a father of a randomly selected case would be $0.334 \times (1\ 20) \times 0.45$, i.e. 0.75%. If account were taken of the reduced fertility of homozygous fathers, this projected value would be revised downwards somewhat. It will be observed that this is similar to the approximate estimate (0.5%) of risk to the father of a case. [Under the alternative assumption (see Demographics section) the projected risk to a father would be 1.6%.]

The assumption was made above that the non-familial proportion was small. This seems reasonable as the allele frequency of 1 20, i.e. 50,000 per $10^6$ is much greater than the gene mutation rates (given earlier) that have been reported for other inherited conditions.

### Discussion

We have argued that, as the fraction of bilateral cases is much larger than that accounted for by chance in a homogeneous population, there must be a predisposed subpopulation. As the distribution of age of onset of the first tumour in bilateral disease is similar to that seen in familial cases, and is unlike that seen in general cases, we have assumed that the predisposition is genetic. The ratio between bilateral and unilateral disease in familial cases is estimated at 0.172 and implies a genetic penetrance of 0.45. Application of this same ratio to the general testis cancer population leads to the estimate that 33.4% of these are hereditary (Figure 2). This in turn allows us to predict the risk to brothers and fathers of cases on the assumption of inheritance of a single predisposing gene. If the predisposing gene is assumed to be inherited in homozygous form the projected risk to brothers and fathers is broadly in line with that reported elsewhere.

Our analysis is based on data which was collected under diverse conditions over many years and depends in part on small numbers of cases. As with any retrospective analysis it is impossible to take account of unsuspected biases which probably are of greater importance than sampling error. The assumptions we have made which underlie the model may be wrong. Thus, it may be that certain unilateral cases are predisposed to a contralateral tumour by some unsuspected biological mechanism, or that there is more than one predisposed subpopulation of individuals, or that the mode of inheritance is of a more complex nature, involving more than one gene or genomic imprinting. However, we base our analysis on the simplest possible set of assumptions which fit the data reasonably well.

If the assumption that the malignant phenotype is a homozygous state is correct, the allele frequency is estimated at about 1 20. This implies that the incidence of heterozygous individuals in the general population would be 1 in 10. It is tempting to speculate that some, or indeed all, of 'sporadic' cases may be in these heterozygous individuals. Thus, these 'sporadic' cases may in fact be the product of a heterozygous predisposition together with a low penetrance, of the order of 0.023 or less. Such a low penetrance would ensure that essentially all such cases would be unilateral and so would not invalidate the assumptions of our analysis. It is of interest that the 129 strain of mice shows just such a pattern of inherited predisposition to testicular teratoma for the *Ter* gene in both homozygous and heterozygous form (Noguchi and Noguchi, 1985).

### Acknowledgements

We thank Dr J Pritchard for initiating our interest, to Professor D Forman and Dr A Michelski for reading the manuscript and for constructive comments, and to Drs LJ Lilly and R Withers for helpful discussions.

### Appendix

If $p$ is the probability of one or more tumours in either side, the probability of no tumour in either side is $(1 - p)^2$, the probability of unilateral tumour is $2p(1 - p)$ and of bilateral tumours is $p^2$. Thus the ratio of probabilities of bilateral to unilateral tumour is $p\ (2 - 2p) = R$, say, and the ratio of probabilities of no tumour to unilateral tumour is $(1 - p)\ (2p) = 1\ (4R)$. These relationships assume statistical independence between tumorigenesis on the two sides, but do not require any other assumption about the nature of the process.

### References

BACH DW, WEISSBACH L AND HARTLAPP JH. (1983). Bilateral testicular tumor. *J of Urol*, **129**, 989-991.

BARTH V AND KRAUSS M. (1989). Bilaterale hodentumoren und die wertigkeit der kontrollsonographie in der nachuntersuchungs-periode. *Z. Urol. Nephrol.*, **82**, 481-485.

CROW JF. (1986). *Basic concepts in population, quantitative, and evolutionary genetics*. WH Freeman and Company: New York.

CSAPO Z, WEISSMULLER J AND SIGEL A. (1987). Sonographie in der Fruherkennung von nicht-palpablen Zweit-Hodentumoren: Eine prospektive Studie. *Urologe*, **A**, 334-338.

DIECKMANN K-P, BOECKMANN W, BROSIG W, JONAS D AND BAUER H-W. (1986). Bilateral testicular germ cell tumors. *Cancer*, **57**, 1245-1258.

DIECKMANN K-P, BECKER T, JONAS D AND BAUER HW. (1987). Inheritance and testicular cancer. *Oncology*, **44**, 367-377.

DIECKMANN K-P, LOY V AND BUTTNER P. (1993). Prevalence of bilateral testicular germ cell tumours and early detection based on contralateral testicular intra-epithelial neoplasia. *Br. J. of Urol.*, **71**, 340-345.

DOLL R, PAYNE P AND WATERHOUSE J. (1966). *Cancer incidence in five continents*, Vol. 1, IUAC Springer-Verlag: Berlin.

EHRENGUT W, SCHWARTAU M AND HUBMANN R. (1980). Testiculare vorerkrankungen bei patienten mit hodentumoren unter besonderer berucksichtigung der mumpsorchitis. *Urologe*, **A19**, 283-288.

EMERY AEH AND MUELLER RF. (1992). *Elements of Medical Genetics*. Churchill Livingstone: Edinburgh.

FORMAN D, OLIVER RTD, BRETT AR, MARSH SGE, MOSES JH, BODMER JG, CHILVERS CED AND PIKE MC. (1992). Familial testicular cancer: a report of the UK family register, estimation of risk and an HLA class I sib-pair analysis. *Br. J. Cancer*, **65**, 255-262.

FOSSA SD AND AASS N. (1989). Cisplatin-based chemotherapy does not eliminate the risk of a second testicular cancer. *Br. J. Urol.*, **63**, 531-534.

GIWERCMAN A, VON DER MAASE H AND SKAKKEBOEK NE. (1993). Epidemiological and clinical aspects of carcinoma in situ of the testis. *Eur. Urol.*, **23**, 104-114.

HENDERSON BE, BENTON B, JING J, YU MC AND PIKE MC. (1979). Risk factors for cancer of the testis in young men. *Int. J. Cancer*, **23**, 598-602.

HOEKSTRA HJ, SLEYFER DT, WOBBES T AND SCHRAFFORDT KOOPS H. (1982). Bilateral primary germ cell tumors of testis. *Urology*, **19**, 152-154.

KNUDSON AG. (1971). Mutation and cancer: statistical study of retinoblastoma. *Proc. Natl Acad. Sci. USA*, **68**, 820-823.

LOY V AND DIECKMANN K-P. (1993). Prevalence of contralateral testicular intraepithelial neoplasia (carcinoma in situ) in patients with testicular germ cell tumour. *Eur. Urol.*, **23**, 120-122.

MUIR C, WATERHOUSE J, MACK T, POWELL J AND WHELAN S. (1987). Cancer incidence in five continents. I.A.R.C. Scientific Publications No. 88. 5: Lyon.

NOGUCHI T AND NOGUCHI M. (1985). A recessive mutation (*ter*) causing germ cell deficiency and a high incidence of congenital testicular teratomas in 129/Sv-*ter* Mice. *J. Natl Cancer Inst.*, **75**, 385-392.

OSTERLIND A, BERTHELSEN JG, ABILGAARD N, HANSEN SO, JENSEN H, JOHANSEN J, MUNCK-HANSEN J AND RASMUSSEN LH. (1987). Incidence of bilateral testicular germ cell cancer in Denmark, 1960-84: preliminary findings. *International Journal of Andrology*, **10**, 203-208.

PATEL SR, RICHARDSON RL AND KVOLS L. (1990). Synchronous and metachronous bilateral testicular tumours. *Cancer*, **65**, 1-4.

PUGH RCB. (1976). Testicular tumours - introduction. In *Pathology of the Testis*, RCB Pugh (ed.) pp. 139-159. Blackwell Scientific Publications: Oxford.

SCHEIBER K, ACKERMANN D AND STUDER UE. (1987). Bilateral testicular germ cell tumors: a report of 20 cases. *J. Urol.*, **138**, 73-76.

STROHMEYER T AND HARTMANN M. (1984). Doppelseitige hodentumoren: fallprasentation und therapiekonzept. *Akt. Urol.*, **15**, 186-189.

THOMPSON J, WILLIAMS CJ, WHITEHOUSE JMA AND MEAD GM. (1988). Bilateral testicular germ cell tumours: an increasing incidence and prevention by chemotherapy. *Br. J. Urol.*, **62**, 374-376.

TOLLERUD DJ, BLATTNER WA, FRASER MC, MORRIS BROWN L, POTTERN L, SHAPIRO E, KIRKEMO A, SHAWKER TH, JAVADPOUR N, O'CONNELL K, STUTZMAN RE AND FRAUMENI JF. (1985). Familial testicular cancer and urogenital developmental anomalies. *Cancer*, **55**, 1849-1854.

VON DER MAASE H, RORTH M, WALBOM-JORGENSEN S, SORENSEN BL, CHRISTOPHERSEN IS, HALD T, JACOBSEN GK, BERTHELSEN JG AND SKAKKEBAEK NE. (1986). Carcinoma in situ of contralateral testis in patients with testicular germ cell cancer: study of 27 cases in 500 patients. *Br. Med. J.*, **293**, 1398-1401.

VON DER MAASE H, GIWERCMAN A, MULLER J AND SKAKKEBAEK NE. (1987). Management of carcinoma-in-situ of the testis. *Int. J. Androl.*, **10**, 209-220.

WAHL C AND HEDINGER C. (1988). Bilaterale keimzelltumoren des hodens. *Schwiz. Med. Wschr.*, **118**, 427-433.

WARE SM, HEYMAN J, AL-ASKARI S AND MORALES P. (1982). Bilateral testicular germ cell malignancy. *Urology*, **19**, 366-372.

WATERHOUSE J, MUIR C, SHANMUGARATNAM K AND POWELL J. (1982). Cancer incidence of five continents. IARC Scientific Publications No. 42. 4. IARC: Lyon.